# Focused Ultrasound Promotes the Delivery of Gastrodin and Enhances the Protective Effect on Dopaminergic Neurons in a Mouse Model of Parkinson’s Disease

**DOI:** 10.3389/fncel.2022.884788

**Published:** 2022-05-17

**Authors:** Yuhong Wang, Kaixuan Luo, Junrui Li, Yehui Liao, Chengde Liao, Wen-Shiang Chen, Moxian Chen, Lijuan Ao

**Affiliations:** ^1^School of Rehabilitation, Kunming Medical University, Kunming, China; ^2^Yunnan Cancer Center, Department of Radiology, Yunnan Cancer Hospital, The Third Affiliated Hospital of Kunming Medical University, Kunming, China; ^3^Department of Physical Medicine and Rehabilitation, National Taiwan University Hospital, National Taiwan University College of Medicine, Taipei City, Taiwan

**Keywords:** focused ultrasound, blood–brain barrier, gastrodin, Parkinson’s disease, drug delivery

## Abstract

Parkinson’s disease (PD) is the second most common chronic neurodegenerative disease globally; however, it lacks effective treatment at present. Focused ultrasound (FUS) combined with microbubbles could increase the efficacy of drug delivery to specific brain regions and is becoming a promising technology for the treatment of central nervous system diseases. In this study, we explored the therapeutic potential of FUS-mediated blood–brain barrier (BBB) opening of the left striatum to deliver gastrodin (GAS) in a subacute PD mouse model induced by 1-methyl-4-phenyl-1,2,3,6-tetrahydropyridine (MPTP). The concentration of GAS in the left hemisphere was detected by ultra-high performance liquid chromatography electrospray Q-Orbitrap mass spectrometry (UHPLC/ESI Q-Orbitrap) and the distribution of tyrosine hydroxylase (TH) neurons was detected by immunohistochemical staining. The expression of TH, Dopamine transporter (DAT), cleaved-caspase-3, B-cell lymphoma 2 (Bcl-2), brain-derived neurotrophic factor (BDNF), postsynaptic density protein 95 (PSD-95), and synaptophysin (SYN) protein were detected by western blotting. Analysis showed that the concentration of GAS in the left hemisphere of PD mice increased by approximately 1.8-fold after the BBB was opened. FUS-mediated GAS delivery provided optimal neuroprotective effects and was superior to the GAS or FUS control group. In addition, FUS enhanced GAS delivery significantly increased the expression of Bcl-2, BDNF, PSD-95, and SYN protein in the left striatum (*P* < 0.05) and reduced the levels of cleaved-caspase-3 remarkably (*P* = 0.001). In conclusion, the enhanced delivery by FUS effectively strengthened the protective effect of GAS on dopaminergic neurons which may be related to the reinforcement of the anti-apoptotic activity and the expression of synaptic-related proteins in the striatum. Data suggests that FUS-enhanced GAS delivery may represent a new strategy for PD treatment.

## Introduction

Parkinson’s disease (PD) is characterized with resting tremor, bradykinesia, rigidity, and postural balance disorders, which is accompanied by non-motor symptoms such as anxiety and depression, and seriously damages patients’ quality of life (Armstrong and Okun, 2020). In 2016, approximately 6.1 million people were diagnosed with PD worldwide, which was about 2.4-fold higher than the number in 1990 (GBD, 2018). Drugs such as carbidopa-levodopa and dopamine agonists can alleviate the dyskinesia caused by early PD; however, their efficacy fluctuates with long-term use, leading to adverse reactions such as dyskinesia and on-off phenomena, thus making it difficult to control the patient’s condition (Armstrong and Okun, 2020).

The blood–brain barrier (BBB) blocks the entry of certain therapeutic drugs into the brain and represents a key obstacle in terms of treating PD. Under physiological conditions, 98% of drugs with a molecular weight of fewer than 400 Daltons and almost 100% of drugs with a molecular weight of more than 500 Daltons cannot pass through the tight junctions of BBB (Pardridge, 2005). Focused ultrasound (FUS) is a new method that could open the BBB and is highly penetrative, non-invasive with good localization and reversibility. In animal and clinical trials, researchers have demonstrated that the combination of FUS and microbubbles can safely as well as reversibly induce the opening of the BBB under specific parameters (Rezai et al., 2020; Wu et al., 2020; Pouliopoulos et al., 2021; Yang et al., 2021). Recently, our team found that the delivery of gastrodin (GAS) *via* FUS-induced BBB opening could improve memory impairment and neuropathology in a mouse model of Alzheimer’s disease (Luo et al., 2022). Moreover, studies have shown that the FUS-mediated delivery of neurotrophic factors and genes can effectively inhibit the rapid progression of neurodegeneration in a mouse model of PD and improve neurological function (Mead et al., 2017; Ji et al., 2019).

Gastrodin, the main active component of *Gastrodia elata*, exerts neuroprotective effects in various neurological diseases. Many studies have confirmed the efficacy of GAS in treating PD (Yan et al., 2019; He et al., 2021). The mechanism of action may involve the anti-oxidation effect (Wang et al., 2014), anti-inflammation effect (Li et al., 2012) and the inhibition of apoptosis (Chen et al., 2017) by GAS. However, only a tiny amount of GAS can enter and disperse throughout the brain in terms of intravenous or oral administration (Lin et al., 2008). Kumar et al. (2013) found that increasing the dose of orally administered GAS could enhance the therapeutic effect of GAS in PD. In another study, Haddadi et al. (2018) attempted to inject GAS into the substantia nigra of rats directly by microinjection; although the concentration of drug increased in specific brain region, the invasive nature of the method was challenging from a clinical point of view. Therefore, it is necessary to identify a non-invasive method to increase the concentration of GAS in specific brain regions without increasing the drug dose as this may represent an effective method to improve the treatment efficiency of PD.

**FIGURE 1:**
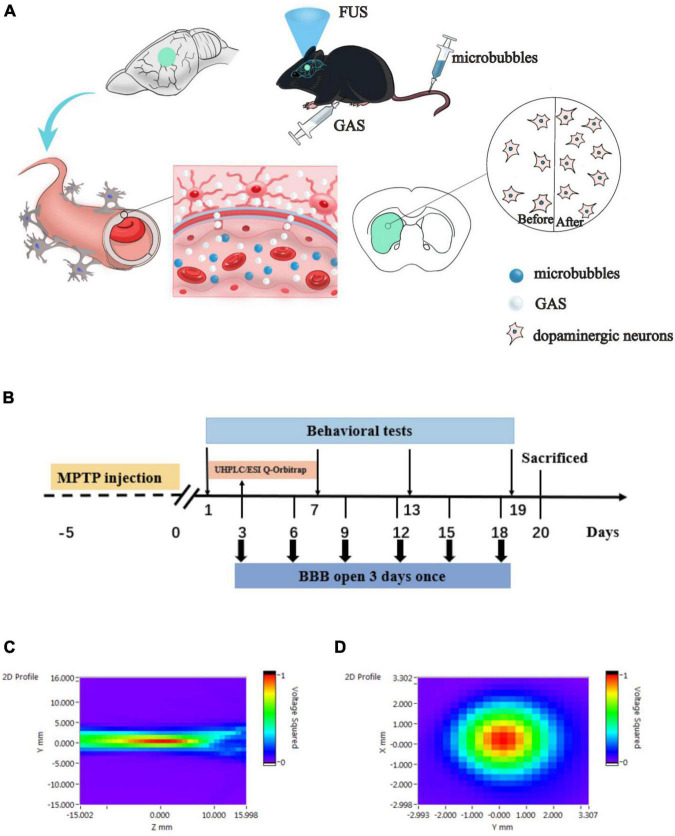
**(A)** Hypothesis of the delivery of gastrodin (GAS) by focused ultrasound (FUS)-induced blood–brain barrier (BBB) opening. FUS induced BBB opening increased the delivery of GAS to specific brain regions in Parkinson’s disease (PD) mice, thereby enhanced neuroprotective effect on dopaminergic neurons. **(B)** Details relating to experimental timing including behavioral testing and FUS operation. MPTP, 1-methyl-4-phenyl-1,2,3,6-tetrahydropyridine; UHPLC/ESI Q-Orbitrap, ultra-high performance liquid chromatography electrospray Q-Orbitrap mass spectrometry. **(C,D)** Acoustic pressure distribution of the lateral and axial direction.

We hypothesize that FUS mediated BBB opening can safely and effectively increase the concentration of GAS in the brain (including the striatum), thus enhancing the protective effect of GAS on dopaminergic neurons (Figure 1A). After confirming that FUS can safely and effectively open the BBB of the left striatum, we established a subacute PD mouse model by injecting 1-methyl-4-phenyl-1,2,3,6-tetrahydropyridine (MPTP). ultra-high performance liquid chromatography electrospray Q-Orbitrap mass spectrometry (UHPLC/ESI Q-Orbitrap) was then used to detect the concentration of GAS in the left hemisphere of the PD mice. Finally, the therapeutic effect of GAS delivered by FUS on PD mice was evaluated by immunohistochemical staining and western blotting.

## Materials and Methods

### Animals

The protocol of this experiment was approved by the Animal Ethics Committee of Kunming Medical University (KMMU2019076). All the experimental procedures followed the guidelines for the care of laboratory animals. All male C57BL/6J mice (8 weeks old, 20–22 g) were purchased from the Experimental Animal Center of Kunming Medical University and housed at 25°C ± 2°C with a fixed 12 h light/dark cycle. All mice had free access to food and water.

### Study Design

In order to test the safety of the BBB opening induced by FUS, healthy C57BL/6J mice were randomly divided into Sham group and FUS output voltage groups of 100, 150, and 200 mV. The opening of the BBB was visualized by Evans blue (EB, Millipore Sigma, Burlington, MA, United States) that exudated from the blood stream, and the safety was verified by hematoxylin-eosin (H&E) as well as Nissl staining. By considering the EB-stained area of the brain section and the results of pathological staining, the optimal FUS parameters to open the BBB were determined. Next, we investigated the efficacy of FUS-induced BBB opening to delivery GAS in PD mice. After adaptation for 2 weeks, 48 male C57BL/6J mice were randomly divided into five subgroups: a Sham group (*n* = 8), an MPTP group (*n* = 8), an MPTP + GAS group (*n* = 12), an MPTP + FUS group (*n* = 8) and an MPTP + FUS + GAS group (*n* = 12). To establish a subacute model of PD on C57BL/6J mice, MPTP (Sigma-Aldrich, St. Louis, MO, United States) was dissolved in saline and subcutaneously injected 30 mg/kg for 5 consecutive days. The Sham group was injected with an equal volume of saline instead. Next, the MPTP + GAS group and the MPTP + FUS + GAS group were intraperitoneally injected with GAS (100 mg/kg/d) for 19 consecutive days; the other groups were injected with saline. FUS sonication was carried out once every 3 days with a total of 6 times. The Sham group, the MPTP group, and the MPTP + GAS group received placebo FUS sonications. Mice in the MPTP + FUS + GAS group were intraperitoneally injected with GAS immediately after the opening of the BBB to facilitate its delivery into the brain. We employed UHPLC/ESI Q-Orbitrap to assess the concentration of GAS in the left hemisphere. Behavioral tests were performed on the 1st, 7th, 13th, and 19th days after MPTP injection to evaluate the motor function of mice. The body weight of the mice was monitored every 3 days once. The mice were sacrificed on the 20th day for relevant biochemical tests (Figure 1B).

### Blood–Brain Barrier Opening Induced by Focused Ultrasound

The FUS sonication system consisted of a waveform generator (RIGOL DG4202, Suzhou, China), a power amplifier (Mini-circuits LZY-22+, New York, United States), and a focused ultrasonic transducer (fundamental frequency: 1 MHz, focal length: 4 cm, diameter: 15 mm). Two-dimensional sound field distributions were measured using a calibrated needle hydrophone (2010, Precision Acoustics, Dorchester, United Kingdom). The lateral and axial sound pressure distributions are shown in Figures 1C,D, which indicates that the ultrasound transducer was well focused. The mice were anesthetized with isoflurane in the animal chamber and then the heads were fixed with prone position. The anesthesia was maintained continuously by inhaled isoflurane with a concentration of 1% through an anesthesia machine (RWD R500, Shenzhen, Guangdong, China). Next, we shaved the fur on the top of the mouse’s head off and removed any remaining fur with hair removal cream. The ultrasonic transducer was placed in an adapter designed for directional FUS delivery and fixed to the left striatum region (0.5 mm front Bregma, 2 mm left). Then, we filled the gap between the mouse scalp and the ultrasound adapter with ultrasound couplant. SonoVue (Bracco Imaging BV, Milan, Italy) microbubbles (1.25 μl/g) were injected into the tail vein. Ten seconds later, the mice were sonicated with FUS. The pulse repetition frequency of FUS was 1 Hz, with a pulse width of 10 ms, and an exposure time of 60 s, output voltage of 100, 150, and 200 mV were used.

### Assessment of Blood–Brain Barrier Disruption and Safety

Twenty-four male C57BL/6J mice were randomly divided into a Sham group (*n* = 6) and the FUS group. The FUS group was further divided into 100, 150, and 200 mV groups (*n* = 6 for each group) to determine the optimal output voltage for FUS-induced BBB opening when other parameters were fixed. Immediately after FUS sonication, 2% of EB dye (6.25 μl/g) was injected through the tail vein. We injected microbubbles and EB dye in the Sham group with placebo FUS sonication. The mice were euthanized after 4 h of EB circulation and then the brain was fixed in paraformaldehyde solution for 24 h (*n* = 3 for each group). Next, we prepared coronal brain slices which covered the center of the ultrasound-sonicated region. The BBB opened area was visualized by EB penetration.

Brain tissues from mice in the Sham and FUS group were collected at 4 h after FUS sonication (*n* = 3 for each group) to test the safety of FUS-induced BBB opening. After dehydration and fixation, tissues were embedded in paraffin and then cut into coronal sections with the thickness of 4 μm. Paraffin sections of the center of sonicated brain region were dewaxed in xylene and anhydrous ethanol and then stained with H&E (Service G1003, Wuhan, Hubei, China). For Nissl staining, the paraffin sections were dewaxed and stained with toluidine blue, differentiated with glacial acetic acid, cleared with xylene, and then sealed with neutral gum. After the optical parameters were determined on normal C57BL/6J mice, we carried out BBB opening of left striatum by FUS for six times with the optimal parameters on PD mice and reassessed the safety by H&E and Nissl staining. Images of the left cortex and striatum in the brain sections were acquired using a dissecting microscope (Olympus, Japan).

### Assessment of Motor Function

#### Pole Test

As previously described, we performed the pole test to assess motor function of mice. A 50-cm–long pole was wrapped with gauze to prevent mice from slipping and a wooden ball was placed on the top (Zhang et al., 2017). The bottom was covered with bedding to protect the mice from fall injury. Mice were trained three times in advance to ensure that all mice could bow their heads down when placed on the ball. Each mouse was placed on the wooden ball and the total time that mouse taken to climb from the top of the pole to the bottom was recorded.

#### Paw Grip Endurance Test

The Paw grip endurance test can evaluate comprehensive motor function, such as muscle strength, muscle tone, and ataxia (Hutter-Saunders et al., 2012). We chose a 45 cm × 30 cm rectangular stainless steel screen, the built-in mesh is 1 cm^2^, and the screen is 25 cm high from the pad. During the test, we placed the mice horizontally on the grid and then quickly flipped the grid 180°, and recorded the time that mice fell off the grid. The upper limit was up to 90 s.

### Detection of Gastrodin Concentration by UHPLC/ESI Q-Orbitrap

We examined GAS concentration in the left hemisphere of mouse after the first time of BBB opening by FUS. Mice in the MPTP + GAS and MPTP + FUS + GAS groups (*n* = 4 for each group) were randomly selected and sacrificed 30 min after the injection of GAS. The tissue of the left hemisphere was separated and placed in a cryotube at −80°C. A standard sample of GAS (G299059-5 g, 62499-27-8, ≥98%, Aladdin, Shanghai, China) was weighed and mixed with pure methanol to prepare a 5.0 mg/ml reserve solution which was then diluted into 1000, 500, 200, 100, and 50 ng/mL standard solutions for analysis and to create a GAS standard curve. The tissue samples were weighed, mixed with methanol, grinded and then centrifuged; the supernatant was taken for testing subsequently. Levels of GAS in the left hemisphere were then quantified with a UltiMate 3000 RS chromatograph (Thermo Fisher Scientific, Waltham, MA, United States) and Q-Exactive high-resolution mass spectrometer (Thermo Fisher Scientific, Waltham, MA, United States). We used a positive electrospray ionization source (ESI); the electrospray voltage was 3.2 kV, the capillary temperature was 300°C, high purity argon was used as the impact gas, the sheath gas was 40 Arb, and the auxiliary gas was 15 Arb. The system was equipped with a Waters T3 150 × 2.1 mm, 3-μm column with an automatic injection volume of 5 μl and a flow rate of 0.30 ml/min. Chromatogram acquisition and the integration of analytes were processed by Xcalibur 4.1 software (Thermo Fisher Scientific, Waltham, MA, United States). Linear regression was performed with a weighting factor of 1/X.

### Immunohistochemistry Staining

In order to evaluate the effects of MPTP administration and different treatments on dopaminergic neurons in the nigrostriatal pathway, we performed tyrosine hydroxylase (TH) immunohistochemical staining to the striatum and substantia nigra brain tissue of each group. Paraffin sections of brain tissue (4-μm-thick) were prepared from each mouse. Following antigen repair and sealing, the sections were incubated with TH antibody (Cat. No. ab137869, dilution 1:500, Abcam, Cambridge, United Kingdom) at 4°C. A biotinylated goat anti-rabbit secondary antibody (Service GB23303, dilution 1:200, Wuhan, China) was added the next day and TH staining was observed under a microscope after 50 min incubation at room temperature.

### Western Blotting

Fresh left striatum and substantia nigra were collected and stored at −80°C for use. The total proteins were extracted by adding radioimmunoprecipitation assay (RIPA) buffer and phenylmethanesulfonyl fluoride (PMSF) protease inhibitor. A bicinchoninic acid (BCA) protein concentration determination kit (Beyotime, China) was used to measure the protein concentration of each sample. Supernatants were dissolved in sample buffer at a protein concentration of 20 μg and separated by 10% sodium dodecyl sulfate-polyacrylamide gel electrophoresis (SDS-PAGE). Separated proteins were then transferred to a polyvinylidene fluoride (PVDF) membrane (Millipore Sigma, Burlington, MA, United States). After blocking with 5% skimmed milk for 2 h, the PVDF membrane was incubated with primary antibodies and placed overnight in a refrigerator at 4°C. The primary antibodies included TH (Cat. No. ab137869, dilution 1:5000, Abcam, Cambridge, United Kingdom), Dopamine transporter (DAT, Cat. No. 22524-1-AP, dilution 1:2000, Proteintech, Rosemont, IL, United States), B-cell lymphoma 2 (Bcl-2, Cat. No. 26593-1-AP, dilution 1:2000, Proteintech, Rosemont, IL, United States), caspase-3 (Cat. No. 19677-1-AP, dilution 1:2000, Proteintech, Rosemont, IL, United States), postsynaptic density protein 95 (PSD-95, Cat. No. 20665-1-Ig, dilution 1:2000, Proteintech, Rosemont, IL, United States), synaptophysin (SYN, Cat. No. 60191-1-Ig, dilution 1:2000, Proteintech, Rosemont, IL, United States), brain-derived neurotrophic factor (BDNF, Cat. No. 66292-1-Ig, dilution 1:2000, Proteintech, Rosemont, IL, United States), GAPDH (Cat. No. 60004-1-Ig, dilution 1:2000, Proteintech, Rosemont, IL, United States), β-Actin (Cat. No. sc-47778, dilution 1:2000, Santa Cruz Biotechnology, Dallas, TX, United States), and α-Tubulin (cat. No. 66031-1-Ig, dilution 1:2000, Proteintech, Rosemont, IL, United States). Following incubation with primary antibodies, the membranes were incubated with anti-rabbit/anti-mouse immunoglobulin G (IgG) enzyme-linked antibody labeled with secondary anti-horseradish peroxidase (HRR-) for 90 min at room temperature. Finally, after washing with TBST, the membranes were developed on a gel developer and the gray values of different protein bands were quantified.

### Statistical Analysis

All data are expressed as mean ± standard error of the mean (SEM). We used SPSS version 25.0 (IBM, Armonk, NY, United States) for statistical analysis. The independent sample *t*-test was used to compare the mean difference between the two groups. One-way ANOVA was used to compare differences in means among the five subgroups. When analysis of variance showed significant differences, pairwise comparisons between means were performed with the least significant difference (LSD) test for *post hoc* analysis. Statistical graphs were generated using GraphPad Prism software version 8.0 (GraphPad Software, Inc., San Diego, CA, United States). Differences were considered to be significant when *P* < 0.05 (bilateral).

## Results

### Efficacy and Safety of Focused Ultrasound-Induced Blood–Brain Barrier Opening

**FIGURE 2:**
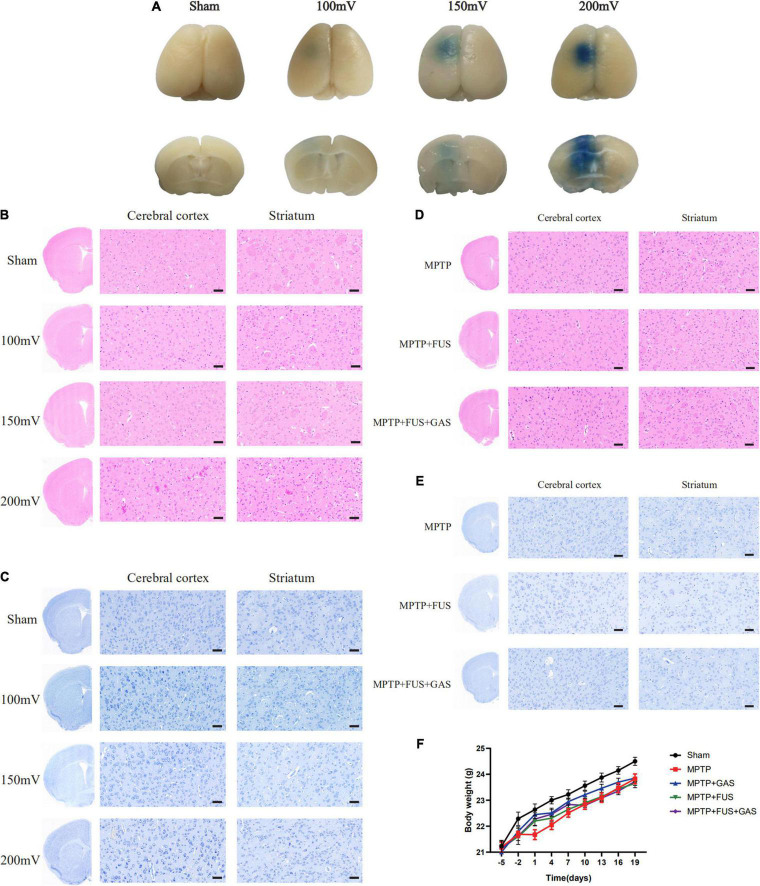
Efficacy and safety of focused ultrasound (FUS)-induced blood–brain barrier (BBB) opening. **(A)** Penetration of Evans blue in the brains of mice in different groups 4 h after FUS-induced BBB opening, *n* = 3. **(B)** H&E staining results of sonicated cerebral cortex and striatum in different groups 4 h after FUS-induced BBB opening, *n* = 3, scale bar = 50 μm. **(C)** Nissl staining results of sonicated cerebral cortex and striatum in different groups 4 h after FUS-induced BBB opening, *n* = 3, scale bar = 50 μm. **(D)** H&E staining results of sonicated cerebral cortex and striatum in Parkinson’s disease (PD) mice after a total of six times’ BBB opening induced by FUS, *n* = 3. Scale bar = 50 μm. **(E)** Nissl staining results of sonicated cerebral cortex and striatum in PD mice after a total of six times’ BBB opening induced by FUS, *n* = 3. Scale bar = 50 μm. **(F)** The body weight change curve of mice in each group.

First, we investigated the efficacy of FUS-induced BBB opening by evaluating the EB penetration. As shown in Figure 2A, all FUS groups were stained by EB, indicating that FUS increased the permeability of the BBB and facilitated EB entry into the brain. However, in the 100 mV group, the penetration depth was shallow and limited to the cortex. There was prominent EB infiltration in the left striatum of the 150 and 200 mV groups, and the EB infiltration area was larger in the 200 mV group. However, H&E staining showed that the sonicated cortex and striatum in the 200 mV group had obvious erythrocyte exudation. In contrast, there was no bleeding in other groups, the cells were evenly arranged, and the nucleoli were normal (Figure 2B). For Nissl staining, the neurons of mice in 100 mV, 150 mV, and Sham group were in good shape and obvious abnormality was absent (Figure 2C). Therefore, 150 mV was selected as the optimal output voltage. After six times’ opening of BBB in PD mice, we again performed histological analysis. Likewise, H&E staining showed no hemorrhage in the ultrasound-sonicated brain area (Figure 2D). The distribution and size of Nissl bodies in the MPTP group and MPTP + FUS group were homogeneous (Figure 2E). The body weight changes of the mice during the experiment are shown in Figure 2F. During the treatment period, there was no statistical difference of body weight between the MPTP + FUS, MPTP + FUS + GAS and the MPTP groups (*P* > 0.05). These data show that it is safe and feasible to use FUS to repeatedly induce the opening of the BBB.

### Focused Ultrasound-Induced Blood–Brain Barrier Opening Increased the Uptake of Gastrodin in the Left Hemisphere of Parkinson’s Disease Mice

**FIGURE 3:**
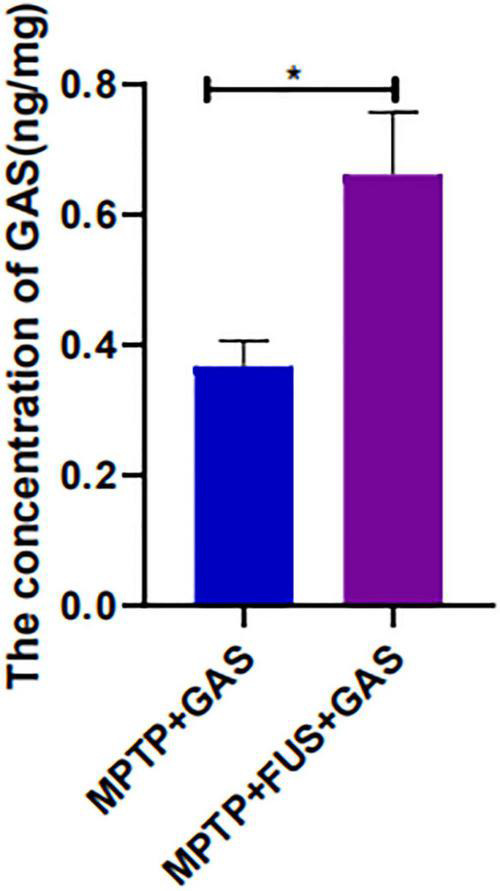
Changes of GAS concentration in the mice’s left hemisphere of the MPTP + GAS group and the MPTP + FUS + GAS group. Compared to the MPTP + GAS group, *P < 0.05; *T*-test, *n* = 4, mean ± SEM.

The concentration of GAS in the left hemisphere was 0.3679 ng/mg in the MPTP + GAS group, After FUS-induced BBB opening, the concentration of GAS increased significantly (*P* = 0.03), reaching to 0.6619 ng/mg, approximately 1.8-fold higher than the MPTP + GAS group (Figure 3).

### Movement Defect Were Induced by 1-Methyl-4-Phenyl-1,2,3,6-Tetrahydropyridine but Recovered 7 Days Later

The climbing time of the pole test in each group was statistically longer than that of the Sham group (*P* < 0.05), while the grid grasping time in the paw grip endurance test was significantly shorter when compared to the Sham group on day 1 (*P* < 0.05). These data suggest that MPTP induced dyskinesia and the PD model was established successfully. The climbing time of the pole test as well as the grid grasping time in the paw grip endurance test between all groups at day 7, 13, and 19 lacked statistical significance (*P* > 0.05), indicating an auto recovery of movement defect (Supplementary Figures 1A,B).

### FUS + GAS Increased the Expression of Tyrosine Hydroxylase and Dopamine Transporter in the Left Nigrostriatal Pathway

**FIGURE 4:**
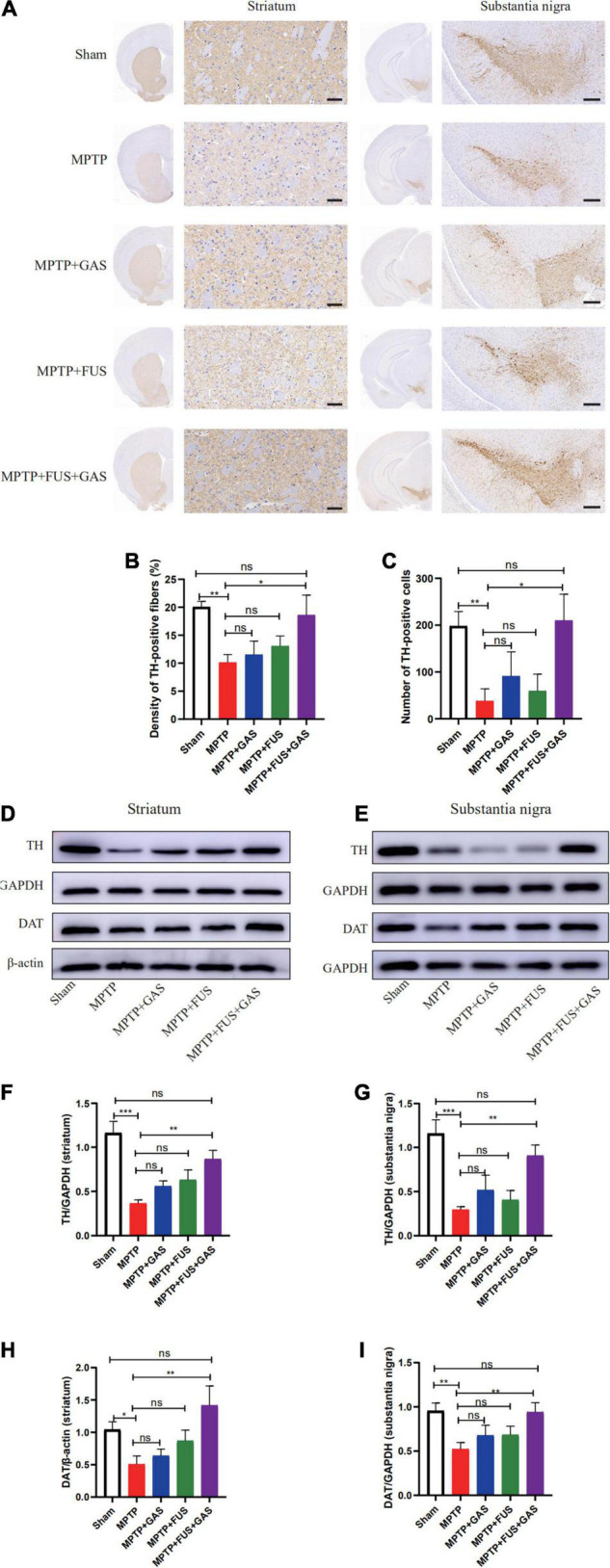
**(A)** Comparison of the immunohistochemical staining of tyrosine hydroxylase (TH)-positive nerve fibers in the left striatum and substantia nigra of mice in each group, *n* = 3. Scale bars = 50 μm for images in stratum; 200 μm for images in substantia nigra. **(B)** Changes of TH-positive nerve fibers in the left striatum of mice in each group. **(C)** Number changes of TH-positive cells in the left substantia nigra of mice in each group. **(D,E)** Representative images of the protein levels of TH and Dopamine transporter (DAT) in the left striatum and substantia nigra of mice in each group. **(F–I)** The left striatum and substantia nigra of mice in each group and changes in the protein expression of TH and DAT. Compared to MPTP group, *P < 0.05; **P < 0.01; ***P < 0.001, no significance (ns), *P* > 0.05. One-way ANOVA with LSD test; *n* = 5, mean ± SEM.

To investigate the effect of FUS + GAS treatment on dopaminergic neurons in the nigrostriatal pathway of PD mice, we used immunohistochemistry assay to stain TH, and the results are shown in Figures 4A–C. Compared with the MPTP group, the density of TH- positive fibers and the number of TH-positive cells in the nigrostriatal pathway of the MPTP + FUS + GAS group recovered significantly (*P* = 0.016). Further qualitative analysis of the protein expression levels of TH and DAT in the left striatum and substantia nigra showed that the protein expression levels of TH and DAT in the MPTP group were lower than the levels of the Sham group (*P* < 0.05). When dealt with FUS + GAS treatment, the protein levels of TH as well as DAT in the left striatum and substantia nigra increased and were significantly higher than those of the MPTP group (*P* < 0.05), while there was no significance (*P* > 0.05) between the Sham group and the FUS + GAS group (Figures 4D–I). Compared with the MPTP group, the number of TH neurons in the left nigrostriatal pathway and the protein expression levels of TH and DAT were increased with varying degrees after FUS or GAS treatment, although there was no statistical significance (*P* > 0.05). Collectively, these results suggest that FUS-induced BBB opening allows the entry of GAS, thus promoting dopamine synthesis and effectively ameliorated MPTP-induced dopaminergic neuron death.

### The Focused Ultrasound Mediated Gastrodin Delivery Enhanced Anti-apoptotic Effects

**FIGURE 5:**
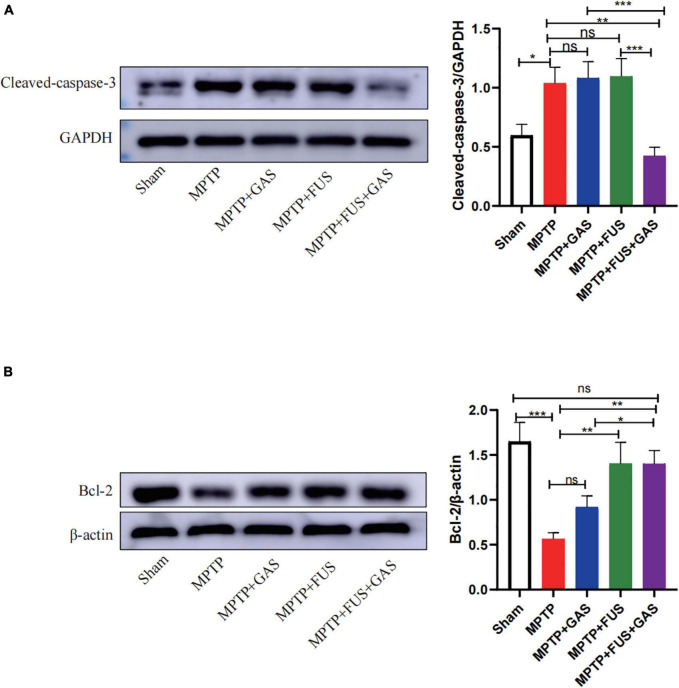
**(A)** Changes in the expression of cleaved-caspase-3 in the left striatum of mice in each group. **(B)** Changes in the expression of B-cell lymphoma 2 (Bcl-2) protein in the left striatum of mice in each group. Compared to MPTP group, *P < 0.05; **P < 0.01; ***P < 0.001, no significance (ns), *P* > 0.05. One-way ANOVA with LSD test; *n* = 5; mean ± SEM.

We found that the level of cleaved-caspase-3 protein in the left striatum after MPTP injection was significantly higher than that of the Sham group (*P* = 0.014) and the level of Bcl-2 significantly decreased (*P* < 0.001), suggesting that the administration of MPTP led to remarkable apoptosis. When dealt with FUS + GAS treatment, the expression level of cleaved-caspase-3 in the left striatum were significantly lower than that of the MPTP group (*P* = 0.001). Furthermore, the expression levels of Bcl-2 were significantly up-regulated by FUS + GAS treatment compared with the MPTP group (*P* = 0.001); the anti-apoptotic effect of FUS + GAS was stronger than GAS treatment alone (*P* < 0.05) (Figures 5A,B).

### Focused Ultrasound Mediated Gastrodin Delivery Upregulated the Expressions of Brain-Derived Neurotrophic Factor, Synaptophysin, and Postsynaptic Density Protein 95

**FIGURE 6:**
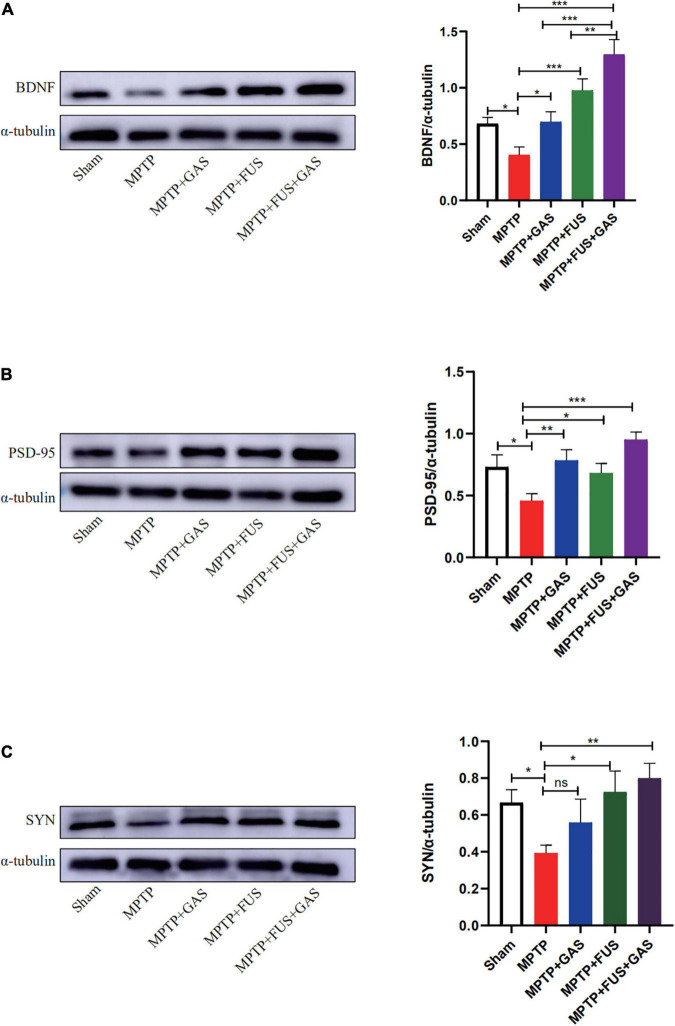
**(A–C)** Changes in the protein expression of brain-derived neurotrophic factor (BDNF), postsynaptic density protein 95 (PSD-95), and synaptophysin (SYN) in the left striatum of mice in each group. Compared to MPTP group, *P < 0.05 compared to MPTP group; **P < 0.01; ***P < 0.001, no significance (ns), *P* > 0.05. One-way ANOVA with LSD test; *n* = 5, mean ± SEM.

Next, we performed western blotting to investigate the effect of FUS + GAS treatment on the expressions of BDNF, SYN, and PSD-95 in the left striatum of PD mice (Figure 6). We found that the protein expression levels of BDNF, SYN, and PSD-95 decreased after the injection of MPTP (*P* < 0.05) while increased after FUS or GAS treatment. The protein levels of BDNF (*P* < 0.001), SYN (*P* < 0.001) and PSD-95 (*P* = 0.003) in the FUS + GAS group were also significantly higher than those of the MPTP group (Figures 6A–C). The BDNF level was higher in FUS + GAS group in comparison of FUS and GAS group (*P* < 0.01). For the expression levels of SYN and PSD-95, the mean values of the FUS + GAS group were higher than that of FUS as well as GAS group, but there was no statistical difference (*P* > 0.05). These results suggest that the delivery of GAS by FUS increased the expression of BDNF, SYN, and PSD-95 in the left striatum of MPTP mice.

## Discussion

In this study, we demonstrated that (1) FUS combined with microbubbles can repeatedly, effectively and non-invasively open the BBB in the striatum of rodents without causing tissue damage. This method also increased the concentration of GAS in the left hemisphere by approximately 1.8-fold; (2) When delivered by FUS, GAS effectively increased the number of dopaminergic neurons in the nigrostriatal pathway; (3) When delivered by FUS, GAS effectively enhanced the anti-apoptotic ability in the striatum and promoted the expression of BDNF and synaptic-related proteins. This study innovatively identified that FUS-induced BBB opening can promote the intraperitoneally injected GAS into the brain and significantly enhances the neuroprotective effect of GAS on dopaminergic neurons in the nigrostriatal pathway.

There are several methods facilitating the delivery of drugs into the brain including intra-arterial infusion of hypertonic solution (Kiviniemi et al., 2017), electroacupuncture stimulation (Zhang et al., 2020), electroporation (Lorenzo et al., 2019), intracranial injection (Liu Y. X. et al., 2018), and intranasal administration (Su et al., 2020), but these methods have obvious limitations. Hypertonic solution and electroacupuncture stimulation open the BBB extensively rather than locally, resulting in the high risk of pathogens and toxic substances to enter the brain. Electroporation and intracranial injection are invasive treatments and face big challenge to repeated operations. Enzymes quickly degrade drugs administered intranasally, and the clearing function of nasal cilia reduces the time that drug get contact with the nasal epithelial cells, which decreases drug absorption. Under appropriate parameters, FUS combined with microbubbles can open the BBB non-invasively, locally, and reversibly thus enhances the delivery of antibodies (Jordão et al., 2010), neurotrophic factors (Lin et al., 2016; Karakatsani et al., 2019), nanoparticles (Ohta et al., 2020) and macromolecular drugs (Park et al., 2017; Wei et al., 2021) to specific brain regions. Due to the drug’s short half-life, it is necessary to open the BBB to deliver the drug repeatedly. Several previous studies reported that when the BBB was continuously opened 8 times in non-human primates at a frequency of every 15 days once, there were no pathological changes in the EEG and somatosensory evoked potentials (Horodyckid et al., 2017). Another research group opened the BBB every 2 days once in rodents and found that repeated BBB opening by FUS at low sound pressure with appropriate microbubbles dose did not cause tissue damage or behavior change. Nevertheless, there were slight and transient behavioral changes when the pressure was significantly higher than required or with excessive microbubbles dose (Tsai et al., 2018). We opened the BBB every 3 days once for a total of 6 times in this experiment. During the experiment, there was no significant difference in mice’s body weight between the FUS group and the MPTP group. At the same time, the pathological sections of the striatum and cortex in the sonicated site showed no abnormality, indicating that the ultrasonic parameters used in this experiment are safe and feasible, exerting great potential to repeated delivery of drugs by FUS.

It was hypothesized that the degeneration of substantia nigra neurons in PD originate from the distal axon (Cheng et al., 2010). It is reported that approximately 30% of the dopamine neurons in the substantia nigra can be damaged while in the striatum it can be as severe as 50–70% in PD (Ma et al., 1997; Cheng et al., 2010). On this basis, the striatum may be an ideal target for PD treatment. MPTP induces PD-related symptoms by damaging dopaminergic neurons and decreasing the density of axons as well as dendrites in the nigrostriatal pathway. Therefore, we selected the MPTP-induced subacute PD model to simulate the symptoms of PD and took striatum as the treatment target. We found that FUS-induced opening of the BBB followed by intraperitoneal injection of GAS significantly increased the number of TH-positive nerve fibers and the expression of TH as well as DAT in the left striatum of PD mice. The concentration of GAS in the left hemisphere increased after FUS-induced BBB opening; the increased concentration enables more GAS to act directly on the damaged dopaminergic neurons. In the study of Ji et al. (2019) FUS was used to open the BBB of the striatum and substantia nigra. The curative effect of the FUS + BDNF group was stronger than that of the FUS group and the BDNF intranasal administration group. Therefore, increasing the release of drugs in the nigrostriatal pathway may be an effective way to improve drug efficacy for the treatment of PD.

Many studies have found that the therapeutic efficacy of GAS is closely related to the therapeutic dose. In terms of AD treatment, 200 mg/kg of GAS was shown to restore the learning and memory ability of AD mice and reduced the deposition of Aβ plaques in the brain; the efficacy of this dose was much higher than that of 100 and 50 mg/kg (Zhou et al., 2016). In another study, Doo et al. (2014) compared the therapeutic effects of GAS at doses of 200, 400, and 800 mg/kg in rats with PD. The improvement of motor function of the 800 mg/kg group was greater than other groups; furthermore, the 800 mg/kg group showed a therapeutic effect earlier than the low-dose group. Although increasing the dose can improve the drug’s efficacy, GAS has a short half-life and is poorly permeable to BBB, which limits its therapeutic effect (Liu Y. et al., 2018). Opening the BBB is a good way to increase the GAS concentration in the brain and to prolong the half-life. It was shown that during the 24 h of BBB opening, GAS in CSF maintained at a high concentration (Kung et al., 2021). In this study, we used FUS to non-invasively as well as effectively enhance the transport of GAS into the left hemisphere, which increased the concentration of GAS in the hemisphere of mice by approximately 1.8 times, effectively improved the therapeutic efficacy of GAS. Compared with the single intraperitoneal injection therapy, FUS induced BBB opening and transferred greater amount of GAS into the brain parenchyma and should reduce the drug consumption by plasma proteins and enzymes. Wang et al. (2009) found that GAS was concentrated in the cortex and cerebellum when entered the brain, and only 4.2% of the drugs could enter the striatum. We used FUS to open the BBB in the striatal region so that the drug can be concentrated in a specific brain region to give play to its effect. Therefore, non-invasive and local BBB opening by FUS may be an effective way to enhance GAS delivery and to reduce the injection dose or frequency of administration to a certain extent and exerts broad prospects for clinical application.

Apoptosis is one of the main causes of dopaminergic neuron death in PD mice. The inhibition of apoptosis has been considered as a potential therapeutic strategy for PD. In the present study, we found that MPTP increased the expression of cleaved-caspase-3 protein in the striatum and decreased the expression of Bcl-2 protein, that is MPTP promoted apoptosis. However, the apoptosis was reversed by FUS + GAS treatment. Therefore, we speculate that the protective effect of FUS + GAS on dopaminergic neurons is partly due to the enhanced anti-apoptotic effect in the striatum. A previous study also found that GAS increased the expression of Bcl-2 and inhibited MPTP-induced caspase-3 activation and protected dopaminergic neurons, in which the anti-apoptotic effect increased as the dosage of GAS increased (Kumar et al., 2013). In the present study, the anti-apoptotic effect of the FUS + GAS group was significantly higher than the GAS group; this may also be related to the increase of GAS concentration after the opening of the BBB.

The reduction of dopamine in the striatum of PD may be related to fiber degeneration or loss and the synaptic reduction in the nigrostriatal pathway, while the effective transport of dopamine is closely related to the integrity of the nigrostriatal pathway (Villalba and Smith, 2018). After injury to the nervous system, the levels of SYN reflect the degree of synaptic remodeling; furthermore, the accumulation of PSD-95 in synapses can promote synaptic maturation as well as excitatory synapse enhancement (Kim et al., 2007). It has been found that MPTP reduces the density of dendritic spines in the striatum of mice and increases the expression of SYN while PSD-95 can restore the density of dendritic spines and relieve the symptoms of PD, at least to some extent (Toy et al., 2014). In addition, the increased expression of SYN and GAP43 can promote axonal regeneration and synaptic remodeling, thereby repairs the damaged dopamine transport pathway and increases dopamine release (Wang et al., 2008). BDNF can promote the maturation and genesis of synapses, and the increased expression of BDNF is essential to the increase of synaptic activity (Lipsky and Marini, 2007; Sen et al., 2016). In the present study, we found that FUS + GAS treatment could increase the expression of BDNF significantly and improve the reduced PSD-95 and SYN level in the PD model induced by MPTP. That is, FUS + GAS may increase the number of terminal synaptic vesicles and the striatal synaptic density of dopaminergic neurons by increasing the expression of BDNF, which favors the enhancement of synaptic transmission, and promotes the release of dopamine. In addition, BDNF is also closely related to the growth and development of dopaminergic neurons (Palasz et al., 2020); therefore, an increase of BDNF may also improve the neuroprotective effect of FUS + GAS.

The pole and paw grip endurance tests are the most used behavioral methods to test MPTP-induced dopamine damage in the substantia nigra and striatum. Previous studies described dyskinesia of subacute PD mice induced by the injection of MPTP (Koppula et al., 2021; Qi et al., 2021). However, some studies have found that dyskinesia is not always apparent in this model, it may even present hyperactivity (Rousselet et al., 2003; Zhang et al., 2017). In the present study, the immunohistochemical staining of mice at the 20 days showed the dopaminergic neurons in the nigrostriatal pathway were severely damaged; however, the impairment of motor function in the experimental mice was not consistent with the pathological manifestations. This may be related to the compensatory ability of the dopaminergic system. It has been found that when the dopamine neurons in the nigra striatum were damaged, the striatum itself has a special compensatory mechanism, that is, the remaining dopaminergic neurons may release more dopamine and result in a significant increase in the proportion of dopamine metabolites in the striatum (Luchtman et al., 2009; Zhang et al., 2017), thus improve the dyskinesia. What’s more, Rousselet et al. (2003) suggested that dopamine in the prefrontal cortex may be transferred to the nigrostriatal system to play a compensatory role. The recovery of activity in MPTP mice may also be related to the increase of norepinephrine content. One study found a significant norepinephrine increase in the striatum of MPTP-induced subacute PD mice, which may lead to overactivity (Rousselet et al., 2003).

There are some limitations in the present study. First of all, the GAS concentration in the hemisphere was detected quantitatively by UHPLC/ESI Q-Orbitrap. However, the distribution and metabolism of GAS in the brain are not clear, which demands further research. Secondly, the dose of GAS (100 mg/kg) that was intraperitoneal injected in this study originates from the past experimental reports in rodents (60–800 mg/kg) (Doo et al., 2014; Wang et al., 2014; Liu Y. et al., 2018). In the view that opening of BBB will increase GAS concentration in the hemisphere of mice, it is necessary to explore the optimal dose of GAS under the appearance of FUS-induced BBB opening in terms of treating PD. Furthermore, the effective maintaining time of GAS in the brain which is delivered by FUS-induced BBB opening need to be further clarified to determine the best frequency of BBB opening induced by FUS.

## Conclusion

In this study, FUS was employed to induce the BBB opening of striatal repetitively and safely in a mouse model of PD, which significantly increased the concentration of GAS in the sonicated hemisphere and effectively promoted the protective effect of GAS on dopaminergic neurons, representing an exciting option for the treatment of PD. The enhanced delivery of GAS by FUS induced BBB opening may provide a promising alternative for the treatment of chronic neurodegenerative diseases.

## Data Availability Statement

The raw data supporting the conclusions of this article will be made available by the authors, without undue reservation.

## Ethics Statement

The animal study was reviewed and approved by the Animal Ethics Committee of Kunming Medical University.

## Author Contributions

LA and MC contributed to the design of the study. YW, KL, and JL carried out the experiment. YW, CL, and W-SC analyzed the data. YW and KL composed the manuscript. All authors contributed to the article and approved the submitted version.

## Conflict of Interest

The authors declare that the research was conducted in the absence of any commercial or financial relationships that could be construed as a potential conflict of interest.

## Publisher’s Note

All claims expressed in this article are solely those of the authors and do not necessarily represent those of their affiliated organizations, or those of the publisher, the editors and the reviewers. Any product that may be evaluated in this article, or claim that may be made by its manufacturer, is not guaranteed or endorsed by the publisher.

## Supplementary Material

Supplementary Figure 1 Behavioral changes of mice in each group at different time points. **(A,B)** The pole climbing time and grip time of mice in each group were compared at different time points after injection of 1-methyl-4-phenyl-1,2,3,6-tetrahydropyridine (MPTP). Compared to the Sham group. ^∗^*P* < 0.05; ^∗∗^*P* < 0.01; ^∗∗∗^*P* < 0.001. One-way ANOVA with LSD test; *n* = 8, mean ± SEM.

## References

[B1] Armstrong M. J. Okun M. S. (2020). Diagnosis and Treatment of Parkinson Disease: A Review. *JAMA* 323 548–560. 10.1001/jama.2019.22360 32044947

[B2] Chen L. Liu X. Wang H. Qu M. (2017). Gastrodin Attenuates Pentylenetetrazole-Induced Seizures by Modulating the Mitogen-Activated Protein Kinase-Associated Inflammatory Responses in Mice. *Neurosci. Bull.* 33 264–272. 10.1007/s12264-016-0084-z 27909971 PMC5567506

[B3] Cheng H. C. Ulane C. M. Burke R. E. (2010). Clinical progression in Parkinson disease and the neurobiology of axons. *Ann. Neurol.* 67 715–725. 10.1002/ana.21995 20517933 PMC2918373

[B4] Doo A. R. Kim S. N. Hahm D. H. Yoo H. H. Park J. Y. Lee H. (2014). Gastrodia elata Blume alleviates L-DOPA-induced dyskinesia by normalizing FosB and ERK activation in a 6-OHDA-lesioned Parkinson’s disease mouse model. *BMC Complement. Altern. Med.* 14:107. 10.1186/1472-6882-14-107 24650244 PMC3994477

[B5] GBD (2018). Global, regional, and national burden of Parkinson’s disease, 1990-2016: a systematic analysis for the Global Burden of Disease Study 2016. *Lancet Neurol.* 17 939–953. 10.1016/S1474-4422(18)30295-3 30287051 PMC6191528

[B6] Haddadi R. Poursina M. Zeraati F. Nadi F. (2018). Gastrodin microinjection suppresses 6-OHDA-induced motor impairments in parkinsonian rats: insights into oxidative balance and microglial activation in SNc. *Inflammopharmacology* 26 1305–1316. 10.1007/s10787-018-0470-4 29616453

[B7] He J. Li X. Yang S. Li Y. Lin X. Xiu M. (2021). Gastrodin extends the lifespan and protects against neurodegeneration in the Drosophila PINK1 model of Parkinson’s disease. *Food Funct.* 12 7816–7824. 10.1039/d1fo00847a 34232246

[B8] Horodyckid C. Canney M. Vignot A. Boisgard R. Drier A. Huberfeld G. (2017). Safe long-term repeated disruption of the blood-brain barrier using an implantable ultrasound device: a multiparametric study in a primate model. *J. Neurosurg.* 126 1351–1361. 10.3171/2016.3.JNS151635 27285538

[B9] Hutter-Saunders J. A. Gendelman H. E. Mosley R. L. (2012). Murine motor and behavior functional evaluations for acute 1-methyl-4-phenyl-1,2,3,6-tetrahydropyridine (MPTP) intoxication. *J. Neuroimmune Pharmacol.* 7 279–288. 10.1007/s11481-011-9269-4 21431472 PMC3392900

[B10] Ji R. Smith M. Niimi Y. Karakatsani M. E. Murillo M. F. Jackson-Lewis V. (2019). Focused ultrasound enhanced intranasal delivery of brain derived neurotrophic factor produces neurorestorative effects in a Parkinson’s disease mouse model. *Sci. Rep.* 9:19402. 10.1038/s41598-019-55294-5 31852909 PMC6920380

[B11] Jordão J. F. Ayala-Grosso C. A. Markham K. Huang Y. Chopra R. Mclaurin J. (2010). Antibodies targeted to the brain with image-guided focused ultrasound reduces amyloid-beta plaque load in the TgCRND8 mouse model of Alzheimer’s disease. *PLoS One* 5:e10549. 10.1371/journal.pone.0010549 20485502 PMC2868024

[B12] Karakatsani M. E. Wang S. Samiotaki G. Kugelman T. Olumolade O. O. Acosta C. (2019). Amelioration of the nigrostriatal pathway facilitated by ultrasound-mediated neurotrophic delivery in early Parkinson’s disease. *J. Control. Release* 303 289–301. 10.1016/j.jconrel.2019.03.030 30953664 PMC6618306

[B13] Kim M. J. Futai K. Jo J. Hayashi Y. Cho K. Sheng M. (2007). Synaptic accumulation of PSD-95 and synaptic function regulated by phosphorylation of serine-295 of PSD-95. *Neuron* 56 488–502. 10.1016/j.neuron.2007.09.007 17988632

[B14] Kiviniemi V. Korhonen V. Kortelainen J. Rytky S. Keinänen T. Tuovinen T. (2017). Real-time monitoring of human blood-brain barrier disruption. *PLoS One* 12:e0174072. eCollection 2017 10.1371/journal.pone.0174072 28319185 PMC5358768

[B15] Koppula S. Alluri R. Kopalli S. R. (2021). Coriandrum sativum attenuates microglia mediated neuroinflammation and MPTP-induced behavioral and oxidative changes in Parkinson’s disease mouse model. *Excli. J.* 20 835–850. 10.17179/excli2021-3668 34177406 PMC8222636

[B16] Kumar H. Kim I. S. More S. V. Kim B. W. Bahk Y. Y. Choi D. K. (2013). Gastrodin protects apoptotic dopaminergic neurons in a toxin-induced Parkinson’s disease model. *Evid. Based Complement Altern. Med.* 2013:514095. 10.1155/2013/514095 23533492 PMC3603713

[B17] Kung Y. Hsiao M. Y. Yang S. M. Wen T. Y. Chen M. Liao W. H. (2021). A single low-energy shockwave pulse opens blood-cerebrospinal fluid barriers and facilitates gastrodin delivery to alleviate epilepsy. *Ultrason. Sonochem.* 78:105730. 10.1016/j.ultsonch.2021.105730 34464899 PMC8408522

[B18] Li C. Chen X. Zhang N. Song Y. Mu Y. (2012). Gastrodin inhibits neuroinflammation in rotenone-induced Parkinson’s disease model rats. *Neural. Regen. Res.* 7 325–331. 10.3969/j.issn.1673-5374.2012.05.001 25774170 PMC4350113

[B19] Lin C. Y. Hsieh H. Y. Chen C. M. Wu S. R. Tsai C. H. Huang C. Y. (2016). Non-invasive, neuron-specific gene therapy by focused ultrasound-induced blood-brain barrier opening in Parkinson’s disease mouse model. *J. Control Rel.* 235 72–81. 10.1016/j.jconrel.2016.05.052 27235980

[B20] Lin L. C. Chen Y. F. Lee W. C. Wu Y. T. Tsai T. H. (2008). Pharmacokinetics of gastrodin and its metabolite p-hydroxybenzyl alcohol in rat blood, brain and bile by microdialysis coupled to LC-MS/MS. *J. Pharm. Biomed. Anal.* 48 909–917. 10.1016/j.jpba.2008.07.013 18757149

[B21] Lipsky R. H. Marini A. M. (2007). Brain-derived neurotrophic factor in neuronal survival and behavior-related plasticity. *Ann. N. Y. Acad. Sci.* 1122 130–143. 10.1196/annals.1403.009 18077569

[B22] Liu Y. Gao J. Peng M. Meng H. Ma H. Cai P. (2018). A Review on Central Nervous System Effects of Gastrodin. *Front. Pharmacol.* 9:24. 10.3389/fphar.2018.00024 29456504 PMC5801292

[B23] Liu Y. X. Liu W. J. Zhang H. R. Zhang Z. W. (2018). Delivery of bevacizumab by intracranial injection: assessment in glioma model. *Onco Targets Ther.* 11 2673–2683. 10.2147/OTT.S159913 29780259 PMC5951223

[B24] Lorenzo M. F. Thomas S. C. Kani Y. Hinckley J. Lee M. Adler J. (2019). Temporal Characterization of Blood-Brain Barrier Disruption with High-Frequency Electroporation. *Cancers (Basel)* 11:E1850. 10.3390/cancers11121850 31771214 PMC6966593

[B25] Luchtman D. W. Shao D. Song C. (2009). Behavior, neurotransmitters and inflammation in three regimens of the MPTP mouse model of Parkinson’s disease. *Physiol. Behav.* 98 130–138. 10.1016/j.physbeh.2009.04.021 19410592

[B26] Luo K. Wang Y. Chen W. S. Feng X. Liao Y. Chen S. (2022). Treatment Combining Focused Ultrasound with Gastrodin Alleviates Memory Deficit and Neuropathology in an Alzheimer’s Disease-Like Experimental Mouse Model. *Neural. Plast.* 2022:5241449. 10.1155/2022/5241449 35069727 PMC8776436

[B27] Ma S. Y. Röyttä M. Rinne J. O. Collan Y. Rinne U. K. (1997). Correlation between neuromorphometry in the substantia nigra and clinical features in Parkinson’s disease using disector counts. *J. Neurol. Sci.* 151 83–87. 10.1016/s0022-510x(97)00100-7 9335015

[B28] Mead B. P. Kim N. Miller G. W. Hodges D. Mastorakos P. Klibanov A. L. (2017). Novel Focused Ultrasound Gene Therapy Approach Noninvasively Restores Dopaminergic Neuron Function in a Rat Parkinson’s Disease Model. *Nano. Lett.* 17 3533–3542. 10.1021/acs.nanolett.7b00616 28511006 PMC5539956

[B29] Ohta S. Kikuchi E. Ishijima A. Azuma T. Sakuma I. Ito T. (2020). Investigating the optimum size of nanoparticles for their delivery into the brain assisted by focused ultrasound-induced blood-brain barrier opening. *Sci. Rep.* 10 18220. 10.1038/s41598-020-75253-9 33106562 PMC7588485

[B30] Palasz E. Wysocka A. Gasiorowska A. Chalimoniuk M. Niewiadomski W. Niewiadomska G. (2020). BDNF as a Promising Therapeutic Agent in Parkinson’s Disease. *Int. J. Mol. Sci.* 21:1170. 10.3390/ijms21031170 32050617 PMC7037114

[B31] Pardridge W. M. (2005). The blood-brain barrier: bottleneck in brain drug development. *NeuroRx* 2 3–14. 10.1602/neurorx.2.1.3 15717053 PMC539316

[B32] Park J. Aryal M. Vykhodtseva N. Zhang Y. Z. Mcdannold N. (2017). Evaluation of permeability, doxorubicin delivery, and drug retention in a rat brain tumor model after ultrasound-induced blood-tumor barrier disruption. *J. Control Rel.* 250 77–85. 10.1016/j.jconrel.2016.10.011 27742444 PMC5384106

[B33] Pouliopoulos A. N. Kwon N. Jensen G. Meaney A. Niimi Y. Burgess M. T. (2021). Safety evaluation of a clinical focused ultrasound system for neuronavigation guided blood-brain barrier opening in non-human primates. *Sci. Rep.* 11:15043. 10.1038/s41598-021-94188-3 34294761 PMC8298475

[B34] Qi H. Shen D. Jiang C. Wang H. Chang M. (2021). Ursodeoxycholic acid protects dopaminergic neurons from oxidative stress *via* regulating mitochondrial function, autophagy, and apoptosis in MPTP/MPP(+)-induced Parkinson’s disease. *Neurosci. Lett.* 741 135493. 10.1016/j.neulet.2020.135493 33181233

[B35] Rezai A. R. Ranjan M. D’haese P. F. Haut M. W. Carpenter J. Najib U. (2020). Noninvasive hippocampal blood-brain barrier opening in Alzheimer’s disease with focused ultrasound. *Proc. Natl. Acad. Sci. U S A* 117 9180–9182. 10.1073/pnas.2002571117 32284421 PMC7196825

[B36] Rousselet E. Joubert C. Callebert J. Parain K. Tremblay L. Orieux G. (2003). Behavioral changes are not directly related to striatal monoamine levels, number of nigral neurons, or dose of parkinsonian toxin MPTP in mice. *Neurobiol. Dis.* 14 218–228. 10.1016/s0969-9961(03)00108-6 14572444

[B37] Sen A. Hongpaisan J. Wang D. Nelson T. J. Alkon D. L. (2016). Protein Kinase Cϵ (PKCϵ) Promotes Synaptogenesis through Membrane Accumulation of the Postsynaptic Density Protein PSD-95. *J. Biol. Chem.* 291 16462–16476. 10.1074/jbc.M116.730440 27330081 PMC4974361

[B38] Su Y. Sun B. Gao X. Dong X. Fu L. Zhang Y. (2020). Intranasal Delivery of Targeted Nanoparticles Loaded With miR-132 to Brain for the Treatment of Neurodegenerative Diseases. *Front. Pharmacol.* 11:1165. 10.3389/fphar.2020.01165 32848773 PMC7424054

[B39] Toy W. A. Petzinger G. M. Leyshon B. J. Akopian G. K. Walsh J. P. Hoffman M. V. (2014). Treadmill exercise reverses dendritic spine loss in direct and indirect striatal medium spiny neurons in the 1-methyl-4-phenyl-1,2,3,6-tetrahydropyridine (MPTP) mouse model of Parkinson’s disease. *Neurobiol. Dis.* 63 201–209. 10.1016/j.nbd.2013.11.017 24316165 PMC3940446

[B40] Tsai H. C. Tsai C. H. Chen W. S. Inserra C. Wei K. C. Liu H. L. (2018). Safety evaluation of frequent application of microbubble-enhanced focused ultrasound blood-brain-barrier opening. *Sci. Rep.* 8 17720. 10.1038/s41598-018-35677-w 30531863 PMC6286368

[B41] Villalba R. M. Smith Y. (2018). Loss and remodeling of striatal dendritic spines in Parkinson’s disease: from homeostasis to maladaptive plasticity? *J. Neural. Transm. (Vienna)* 125 431–447. 10.1007/s00702-017-1735-6 28540422 PMC5701884

[B42] Wang Q. Chen G. Zeng S. J. (2009). Study on the Metabolism of Gastrodin in Rat Brain, Liver, Kidney and Different Brain Regions Homogenate. *Chin. J. Mod. Appl. Pharm.* 26 614–619.

[B43] Wang Q. D. Gu P. Dong Q. Y. (2008). The effect of repetitive transcranial magnetic stimulation on the expressions of growth associated protein-43 and synaptophysin in striatum of Parkinson’s disease model mice. *Chin. J. Gerontol.* 19, 1885–1888.

[B44] Wang X. L. Xing G. H. Hong B. Li X. M. Zou Y. Zhang X. J. (2014). Gastrodin prevents motor deficits and oxidative stress in the MPTP mouse model of Parkinson’s disease: involvement of ERK1/2-Nrf2 signaling pathway. *Life Sci.* 114 77–85. 10.1016/j.lfs.2014.08.004 25132361

[B45] Wei H. J. Upadhyayula P. S. Pouliopoulos A. N. Englander Z. K. Zhang X. Jan C. I. (2021). Focused Ultrasound-Mediated Blood-Brain Barrier Opening Increases Delivery and Efficacy of Etoposide for Glioblastoma Treatment. *Int. J. Radiat. Oncol. Biol. Phys.* 110 539–550. 10.1016/j.ijrobp.2020.12.019 33346092 PMC8553628

[B46] Wu S. K. Tsai C. L. Huang Y. Hynynen K. (2020). Focused Ultrasound and Microbubbles-Mediated Drug Delivery to Brain Tumor. *Pharmaceutics* 13.:15. 10.3390/pharmaceutics13010015 33374205 PMC7823947

[B47] Yan J. Yang Z. Zhao N. Li Z. Cao X. (2019). Gastrodin protects dopaminergic neurons *via* insulin-like pathway in a Parkinson’s disease model. *BMC Neurosci.* 20:31. 10.1186/s12868-019-0512-x 31208386 PMC6580469

[B48] Yang Q. Zhou Y. Chen J. Huang N. Wang Z. Cheng Y. (2021). Gene Therapy for Drug-Resistant Glioblastoma *via* Lipid-Polymer Hybrid Nanoparticles Combined with Focused Ultrasound. *Int. J. Nanomedicine.* 16 185–199. 10.2147/IJN.S286221 33447034 PMC7802796

[B49] Zhang Q. S. Heng Y. Mou Z. Huang J. Y. Yuan Y. H. Chen N. H. (2017). Reassessment of subacute MPTP-treated mice as animal model of Parkinson’s disease. *Acta. Pharmacol. Sin.* 38 1317–1328. 10.1038/aps.2017.49 28649132 PMC5630672

[B50] Zhang S. Gong P. Zhang J. Mao X. Zhao Y. Wang H. (2020). Specific Frequency Electroacupuncture Stimulation Transiently Enhances the Permeability of the Blood-Brain Barrier and Induces Tight Junction Changes. *Front. Neurosci.* 14:582324. 10.3389/fnins.2020.582324 33122995 PMC7573286

[B51] Zhou N. N. Zhu R. Zhao X. M. Zhang J. M. Liang P. (2016). [Effect and mechanism of gastrodin inhibiting β-amyloid plaques in brain of mice]. *Yao. Xue. Xue. Bao.* 51 588–594. 29859528

