# Supplementary figures and images for: Focused Ultrasound Promotes the Delivery of Gastrodin and Enhances the Protective Effect on Dopaminergic Neurons in a Mouse Model of Parkinson’s Disease
Source: Front Cell Neurosci. 2022 May 17;16:884788. doi: 10.3389/fncel.2022.884788 (PMC9152004; doi:10.3389/fncel.2022.884788)

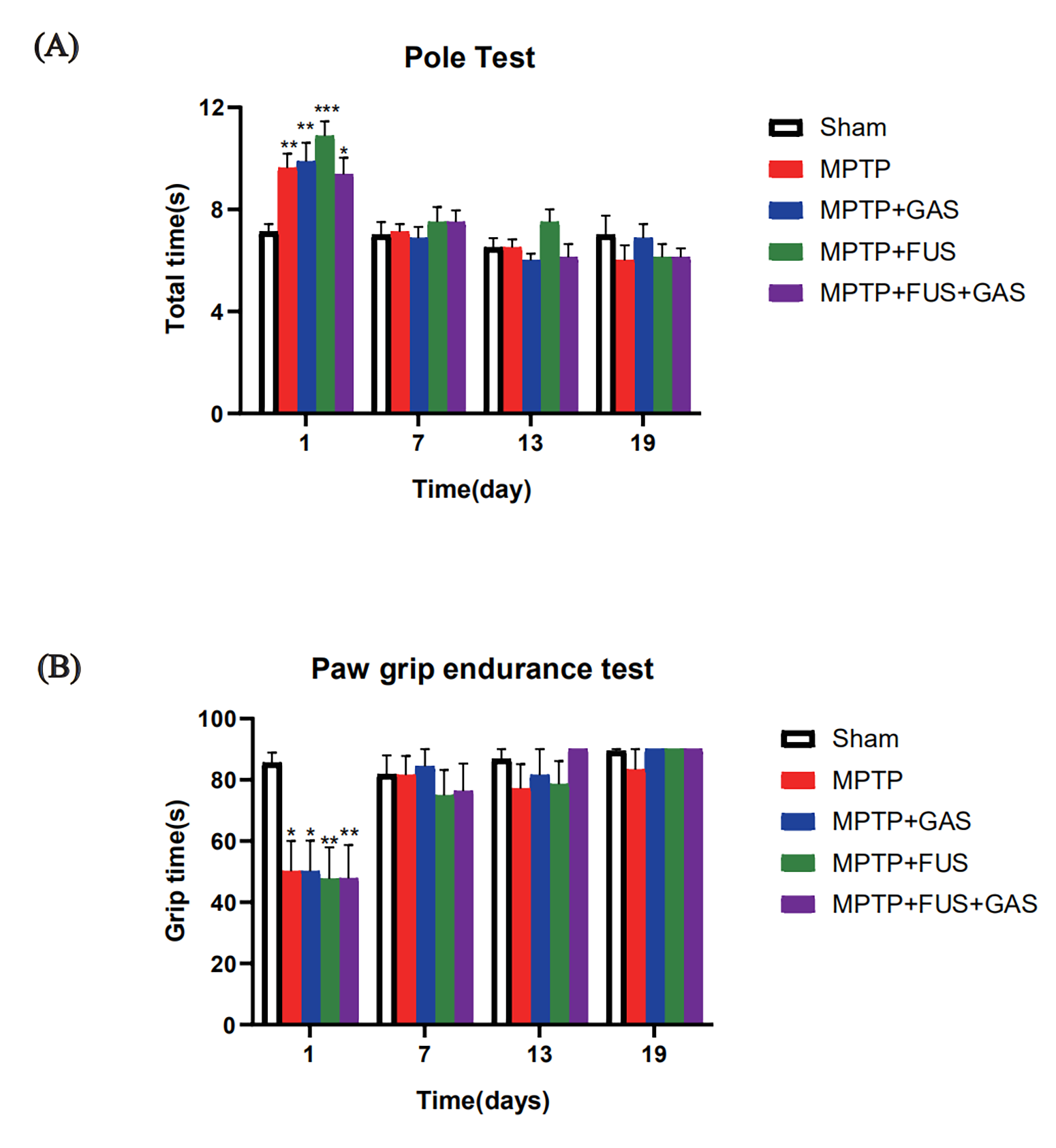

Supplement: Supplementary Figure 1 — Behavioral changes of mice in each group at different time points. (A,B) The pole climbing time and grip time of mice in each group were compared at different time points after injection of 1-methyl-4-phenyl-1,2,3,6-tetrahydropyridine (MPTP). Compared to the Sham group. ∗P < 0.05; ∗∗P < 0.01; ∗∗∗P < 0.001. One-way ANOVA with LSD test; n = 8, mean ± SEM. [file Image_1.TIF]
